# Association of metabolic and genetic heterogeneity in head and neck squamous cell carcinoma with prognostic implications: integration of FDG PET and genomic analysis

**DOI:** 10.1186/s13550-019-0563-0

**Published:** 2019-11-21

**Authors:** Jinyeong Choi, Jeong-An Gim, Chiwoo Oh, Seunggyun Ha, Howard Lee, Hongyoon Choi, Hyung-Jun Im

**Affiliations:** 1 Department of Transdisciplinary Studies, Graduate School of Convergence Science and Technology, Seoul National University, Seoul, Republic of Korea; 2 Radiation Medicine Research Institute, Seoul National University College of Medicine, Seoul, Republic of Korea; 3 Department of Nuclear Medicine, Seoul ST. Mary’s Hospital, Seoul, Republic of Korea; 4 Department of Clinical Pharmacology and Therapeutics, Seoul National University College of Medicine and Hospital, Seoul, Republic of Korea; 5 Department of Nuclear Medicine, Seoul National University Hospital, Seoul, Republic of Korea

**Keywords:** ^18^F-fluorodeoxyglucose, Positron emission tomography, Heterogeneity, Radiogenomics, MATH

## Abstract

**Purpose:**

The linkage between the genetic and phenotypic heterogeneity of the tumor has not been thoroughly evaluated. Herein, we investigated how the genetic and metabolic heterogeneity features of the tumor are associated with each other in head and neck squamous cell carcinoma (HNSC). We further assessed the prognostic significance of those features.

**Methods:**

The mutant-allele tumor heterogeneity (MATH) score (*n* = 508), a genetic heterogeneity feature, and tumor glycolysis feature (GlycoS) (*n* = 503) were obtained from the HNSC dataset in the cancer genome atlas (TCGA). We identified matching patients (*n* = 33) who underwent 18F-fluorodeoxyglucose positron emission tomography (FDG PET) from the cancer imaging archive (TCIA) and obtained the following information from the primary tumor: metabolic, metabolic-volumetric, and metabolic heterogeneity features. The association between the genetic and metabolic features and their prognostic values were assessed.

**Results:**

Tumor metabolic heterogeneity and metabolic-volumetric features showed a mild degree of association with MATH (*n* = 25, ρ = 0.4~0.5, *P* < 0.05 for all features). The patients with higher FDG PET features and MATH died sooner. Combination of MATH and tumor metabolic heterogeneity features showed a better stratification of prognosis than MATH. Also, higher MATH and GlycoS were associated with significantly worse overall survival (*n* = 499, *P* = 0.002 and 0.0001 for MATH and GlycoS, respectively). Furthermore, both MATH and GlycoS independently predicted overall survival after adjusting for clinicopathologic features and the other (*P* = 0.015 and 0.006, respectively).

**Conclusion:**

Both tumor metabolic heterogeneity and metabolic-volumetric features assessed by FDG PET showed a mild degree of association with genetic heterogeneity in HNSC. Both metabolic and genetic heterogeneity features were predictive of survival and there was an additive prognostic value when the metabolic and genetic heterogeneity features were combined. Also, MATH and GlycoS were independent prognostic factors in HNSC; they can be used for precise prognostication once validated.

## Introduction

Cancer is a heterogenous disease at genetic, epigenetic, and phenotypic levels [1]. Cancer progression is driven by a genetic process of clonal evolution, which eventually causes tumor genetic heterogeneity, a tumor with multiple subsets of subclonal mutations [1]. Acquired tumor genetic heterogeneity is caused by the selective pressures during the evolution process and affected by tumor vasculature and immune system in the microenvironment [2]. Furthermore, genetic heterogeneity eventually drives the phenotypic heterogeneity of tumor by interacting environmental factors [3]. Heterogeneous subsets of tumor have different molecular targets, which may result in different levels of resistance to the cancer treatment [4]. Accordingly, tumor heterogeneity is associated with the progression and eventual clinical outcomes of cancer patients [5]. Thus, evaluation of tumor heterogeneity is crucial for selecting anticancer strategies and predicting clinical outcomes [6]. Advances in next-generation sequencing (NGS) have allowed for extensive understanding of tumor genetic heterogeneity and provided useful features to evaluate of tumor heterogeneity [7, 8]. The mutant allele tumor heterogeneity (MATH), a genetic heterogeneity feature, is easily calculated as a percentage of mutant allele frequencies among tumor-specific mutated loci. MATH has been known to have a prognostic value in HNSC and colon cancer [9, 10].

Phenotypical heterogeneity can be noninvasively studied using various imaging techniques including computed tomography (CT), magnetic resonance imaging (MRI), and ^18^F-fluorodeoxyglucose positron emission tomography (FDG PET) [11]. FDG PET is a compelling image modality to evaluate metabolic heterogeneity of tumors, a phenotypic tumor heterogeneity [12]. Recently, heterogeneity parameters obtained using FDG PET have been extensively evaluated and reported to have diagnostic and prognostic values in multiple types of malignancies including HNSC, non-small cell lung cancer, and pancreatic cancer [12–16]. Although the metabolic features evaluated by FDG PET are closely associated with biological factors in the tumor microenvironment [12, 17, 18], it is still unknown whether metabolic heterogeneity is associated with genetic heterogeneity [14].

Herein, we investigated if metabolic heterogeneity based on FDG PET was associated with genetic heterogeneity represented by MATH. Furthermore, we explored the prognostic value of both metabolic and genetic heterogeneity features in predicting the outcomes of patients with HNSC.

## Materials and methods

### Data acquisition

Genomic and clinical data were obtained from the head and neck squamous cell carcinoma dataset of the cancer genome atlas (TCGA-HNSC). The FDG PET data of the patients included in TCGA-HNSC was obtained from cancer imaging archive (TCIA) which is a publicly available repository. TCGA and TCIA data were acquired by a publicly available dataset that removed patient identifiers. The publicly available data were collected with patients’ informed consent approved by the institutional review boards of all participating institutions following the 1964 Helsinki declaration and its later amendments or comparable ethical standards. A total of 528 clinical information which was updated at 2018/08/30 were acquired National Cancer Institute database for the survival analysis. The somatic variants data were acquired for 508 TCGA-HNSC patients from the NCI database using R data package ‘TCGAmutations.’ Glycolysis signature (GlycoS) was previously assessed for the metabolic signatures of all TCGA samples [19] and downloaded from the website (http://choih.shinyapps.io/metabolicsignatures). In brief, GlycoS data were obtained by RNA sequencing data of TCGA samples by using gene set enrichment analysis and metabolic pathway genes of Reactome [20]. A total of 192 patients of TCGA-HNSC images in the TCIA matched with the genomic data of the TCGA were available. Finally, we identified 33 cases which included baseline FDG PET/CT scans and utilized the FDG PET scans for further analysis.

### Tumor metabolic features analyzed by FDG PET

In this study, primary tumor segmentation of all FDG PET examinations was computed using PETedge tool of MIMvista (MIM Software Inc., USA) by an expert. Before calculating the texture feature, we have changed *X*, *Y*, *Z* values of all data sets as the same values (4.7, 4.7, 3.3) through trilinear voxel interpolation to compare each other. Then feature extraction was performed using LIFEx (IMIV, CEA, France) based on these regions of interest (ROIs) [21]. Maximum standardized uptake value (SUVmax), peak standardized uptake value (SUVpeak), metabolic tumor volume (MTV), and total lesion glycolysis (TLG) are the conventional metabolic or metabolic-volumetric parameters which are the most extensively studied in the previous studies and found to be prognostic in head and neck cancer [22–24]. Among the many heterogeneity parameters, we selected entropy and coefficient of variation (COV) because these features were reproducible and robust values in different reconstruction and acquisition time settings according to previous studies [25, 26]. Two tumor metabolic features (SUVmax and SUVpeak), two metabolic-volumetric (TLG and MTV), and two metabolic heterogeneity features (entropy and COV) were obtained. Entropy was calculated based on SUV histogram using the equation; $\mathrm{Entropy}=-\sum \limits_ip(i)\times {\log}_2\left(p(i)+\varepsilon \right)$. Entropy reflects the randomness of the distribution where p(i) is the probability of occurrence of voxels with intensity *i* and ε = 2e-16. COV was calculated as standard deviation divided by SUVmean of the ROI. There were small tumors among the patients (range 0.36~ 3.93 mL, median 1.14 mL). However, we could calculate entropy and COV in all tumors, because entropy was the histogram-based parameter and COV can be calculated from SUV distribution. Twenty-five head and neck squamous cell carcinoma (HNSC) cases which have both FDG PET feature extraction data and gene mutation data were available to perform correlation analysis.

### Tumor genetic heterogeneity

The MATH score was obtained as a percentage of the median absolute deviation (MAD) and median by clustering the variant allele frequency in each mutated loci using the R ‘maftools’ package [27]. Each MATH score was calculated using MAF files for a total of 508 tumor samples, and this was used for survival analysis together with survival data.

### Survival analysis

Overall survival (OS) can be obtained from the clinical data, which is defined as the period from the date of diagnosis until the date of death from any cause. The censored time is from date of initial diagnosis until the date of last contact (largest number of days) from all the clinical data files [28]. For evaluation of prognostic value of the features, we divided the patients into two groups (high and low groups) according to an optimized cut-off of each feature. The optimized cut-off was selected using the ‘cutoff finder (http://molpath.charite.de/cutoff/index.jsp).’ We selected the method for ‘Survival: significance (log-rank test)’ for cutoff determination. This cutoff is the most significant point from log-rank test which divide the variables into two groups. The high and low groups were compared using the log-rank test and Kaplan Meyer analysis. Cox regression analysis was also performed in multivariable survival analysis using continuous MATH, GlycoS, age, and categorical clinicopathologic variables (sex and tumor stage).

### Statistical analysis

Association between these genomic and tumor metabolic features was analyzed by using correlation analysis and the prognostic value of the parameters were assessed using the log-rank test. To analyze the correlation between two genomic and selected six FDG PET features, Spearman correlation analysis was performed. Correlation coefficients and *p* values were gained and used to sort statistically significant features (*P* value < 0.05). All statistical analyses were performed in R (version 3.4.4) and SPSS (version 25). All tests were two-sided and *P* values less than 0.05 were considered significant.

## Results

### Patient characteristics

**Fig. 1:**
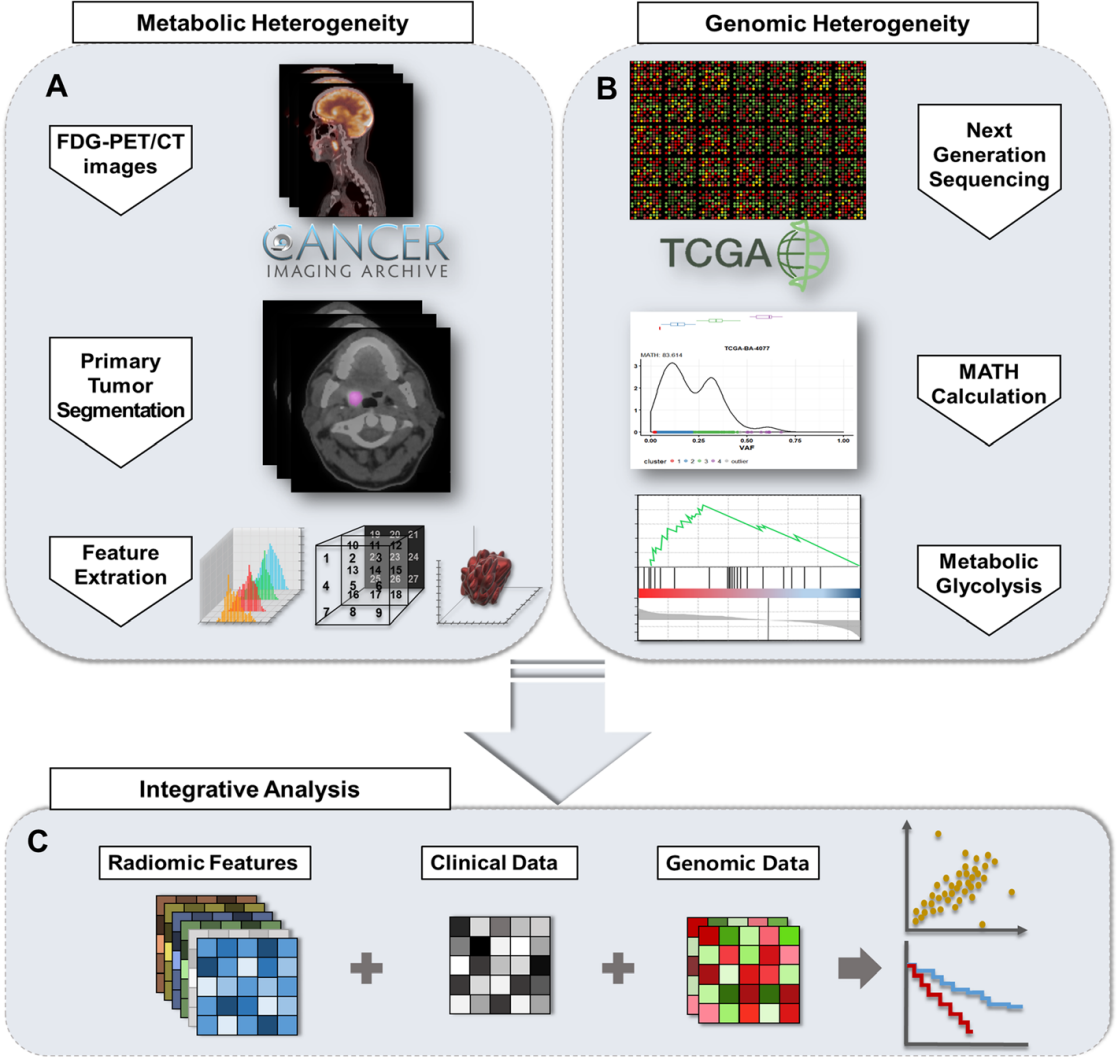
Study scheme. A scheme for integrative study of radiogenomics. FDG PET data and genomic mutation data for TCGA-HNSC dataset were obtained from each database of TCIA and TCGA. **a** The primary tumor was manually assigned, and then ROIs were computed for feature extraction. **b** MATH calculation using MAF files were done in R. Also, metabolic glycolysis (GlycoS) value was obtained using gene set enrichment analysis. **c** Clinical data of TCGA-HNSC was gained from TCGA. Total six features were selected and used for radiogenomic analysis. We statistically analyzed radiomic, clinical and genomic data using correlation analysis, Kaplan-Meier analysis, log-rank test

The scheme of this study is demonstrated in Fig. 1. The number of patients with genomic data was 508. The patients had a median age of 61 years (range of 20–90 years) and a median follow-up days of 633 days (range of 2–6417 days). Among them, 220 patients died during the follow-up. In all patients, 78% were stage III/IV, and there were an about three times higher number of men than women (371 vs. 137).

The characteristics of 25 patients who were available for both genomic and FDG PET analysis are summarized in Table 1. Primary tumor segmentation and feature extraction were performed using FDG PET scans of the patients. The median age of the patients was 56 years (range of 38–84 years). Among 25 patients with a median follow-up of 458 days (range of 30–6417 days), six patients died while 18 were alive.

**Table 1:** Patients characteristics (FDG-PET)

Patients, *n*	25 (1 of not available clinical data)
Median follow-up (days)	458 (30–6417)
Vital status	
Dead	6
Alive	18
Age (years)	
Median	56
Range	38~84
Gender	
Male	18
Female	6
Clinical stage	
I	3
II	5
III	2
IVA	13
IVB	1
IVC	0
Tumor site	
Alveolar ridge	1
Base of tongue	2
Larynx	5
Oral cavity	4
Oral tongue	3
Tonsil	9

### Association between genetic and FDG PET features

**Fig. 2:**
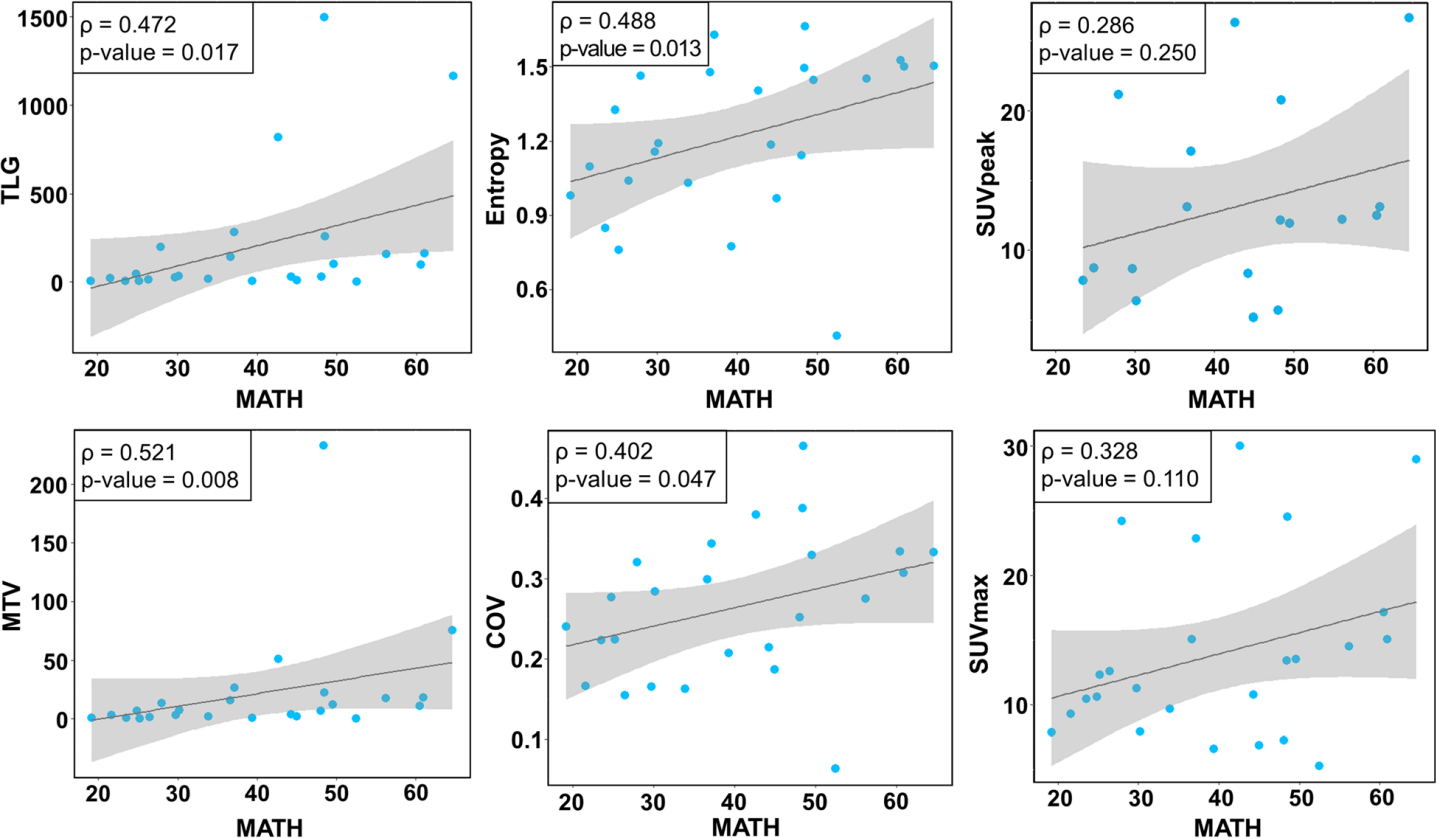
Correlation between MATH and FDG PET features. Scatter plots for correlation analysis of MATH and FDG PET features. Each blue dots represent patients available for MATH and radiomic data (*N* = 25). Upper left box shows Spearman correlation coefficient (ρ) and *P* value. The dark gray line means a linear regression line and the gray region is 95% confidence region

Correlation analyses between MATH, GlycoS, and FDG PET features (SUVmax, SUVpeak, TLG, MTV, entropy, and COV) were performed in 25 patients. Metabolic heterogeneity features and metabolic-volumetric features showed a trend for association with the genetic heterogeneity feature, MATH (ρ = 0.488, *P* = 0.013 for entropy; ρ = 0.402, *P* = 0.047 for COV, ρ = 0.521, *P* = 0.008 for MTV; ρ = 0.472, *P* = 0.017 for TLG) (Figs. 2 and 4). On the other hand, SUVmax and SUVpeak were not significantly associated with MATH (ρ = 0.328, *P* = 0.110 for SUVmax; ρ = 0.286, *P* = 0.250 for SUVpeak) (Fig. 2).

**Fig. 3:**
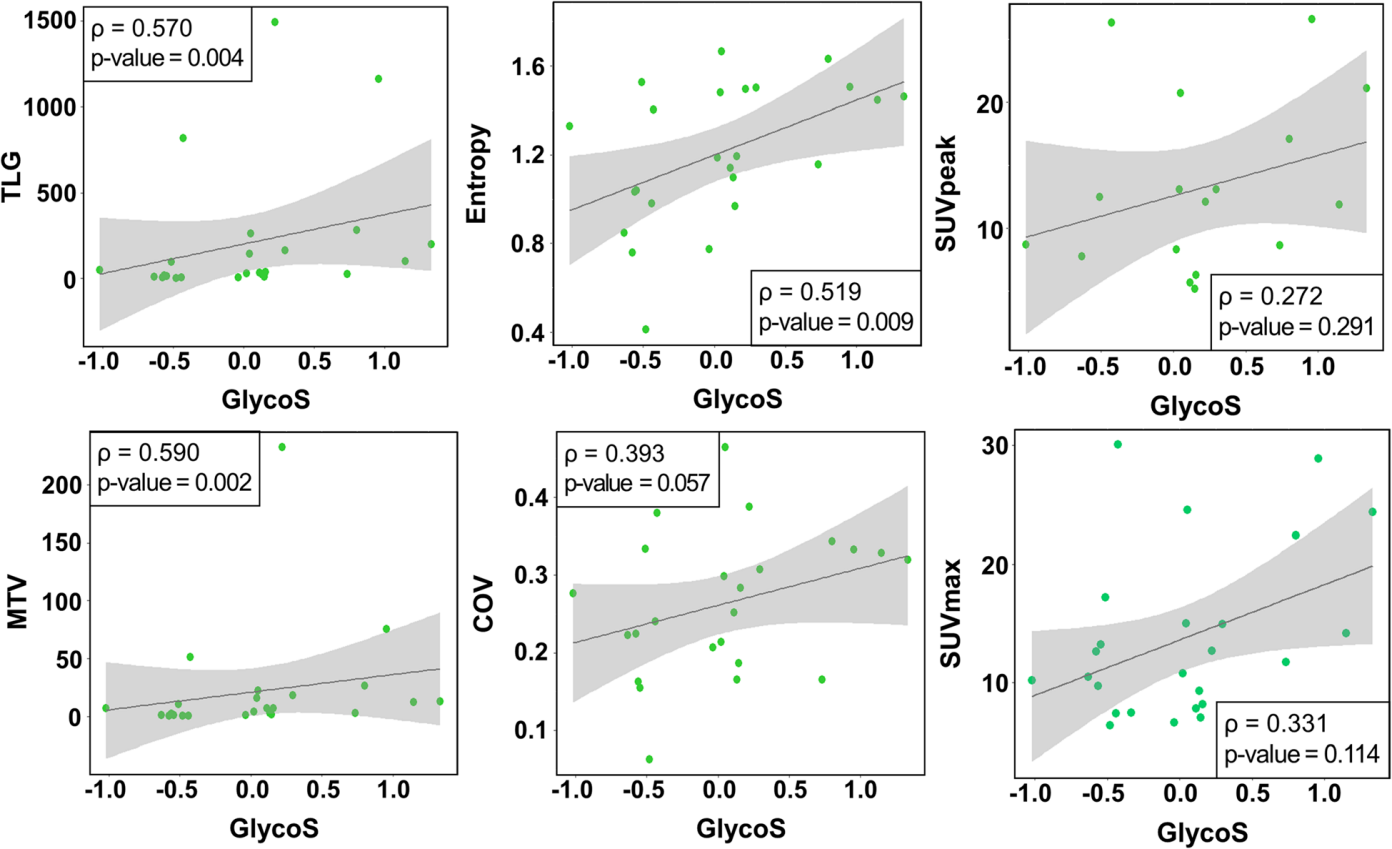
Correlation between GlycoS and FDG PET features. Scatter plots for correlation analysis of GlycoS and FDG PET features. Each green dots represent patients available for MATH and radiomic data (*N* = 25). Upper left box shows Spearman correlation coefficient (ρ) and *P* value. The dark gray line means a linear regression line and the gray region is 95% confidence region

We evaluated the association between GlycoS calculated by gene expression and FDG PET features to use GlycoS as a surrogate of tumor metabolic features for more patients in survival analysis. TLG and MTV from FDG PET showed moderate degree of associations with the GlycoS (ρ = 0.590, *P* = 0.002 for MTV; ρ = 0.570, *P* = 0.004 for TLG). The GlycoS showed a trend of positive correlation with entropy, while COV, SUVmax, and SUVpeak did not (ρ = 0.519, *P* = 0.009 for entropy; ρ = 0.393, *P* = 0.057 for COV; ρ = 0.331, *P* = 0.114 for SUVmax; ρ = 0.272, *P* = 0.291 for SUVpeak) (Fig. 3).

### Prognostic value of the genetic and FDG PET features

**Fig. 4:**
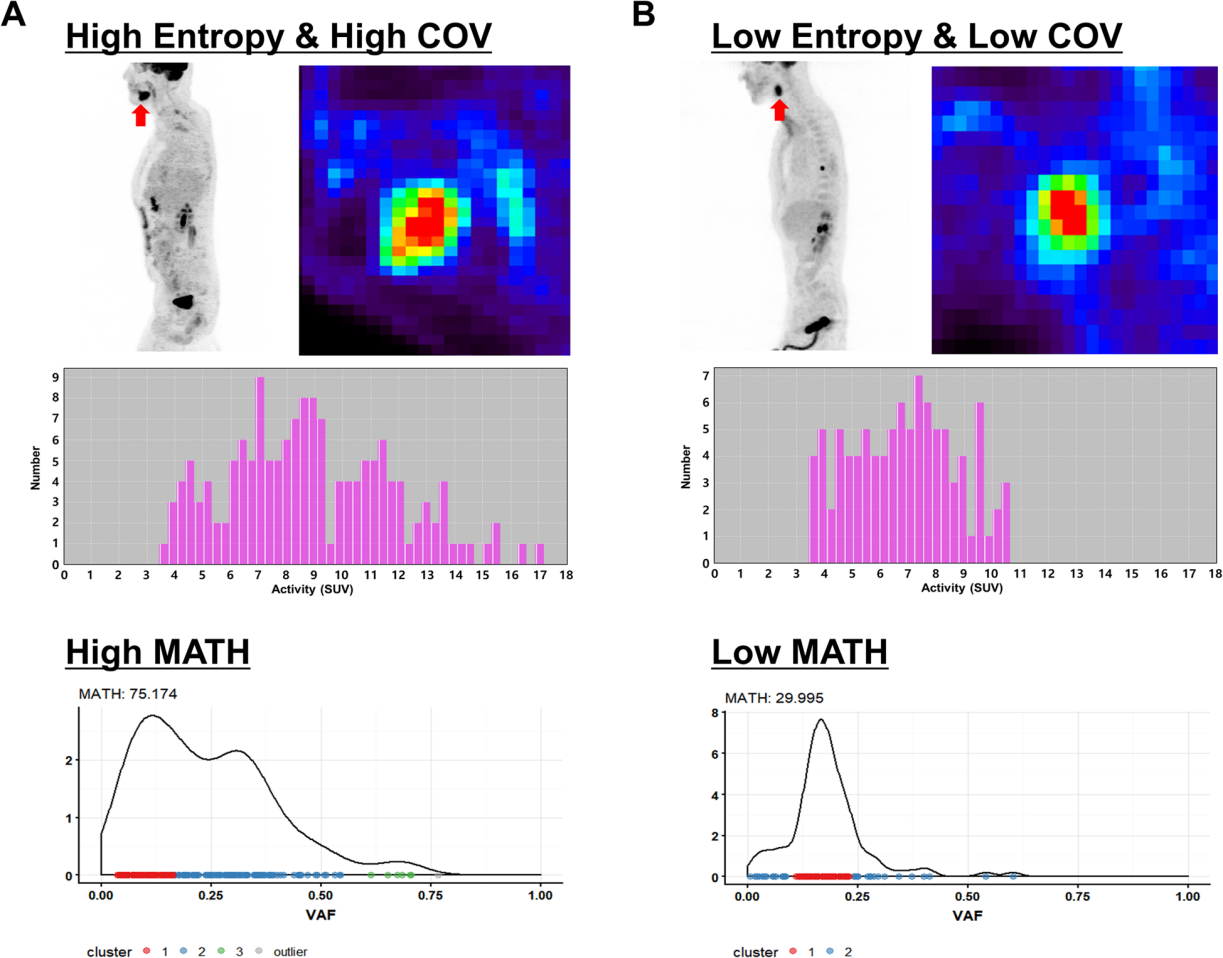
Representative cases. **a** A patient had a tongue cancer with high metabolic heterogeneity (high entropy and high COV groups). Genomic analysis of the patient revealed that the tumor had high genetic heterogeneity (high MATH group). **b** A patient had a left tonsillar cancer with low metabolic heterogeneity (low entropy and low COV group) based on FDG PET. Genomic analysis of the patient revealed that the tumor had low genetic heterogeneity (low MATH group). Of note, low and high entropy, COV, and MATH groups are divided according to the optimized cut-offs obtained by cut-off finder (http://molpath.charite.de/cutoff/index.jsp)

**Fig. 5:**
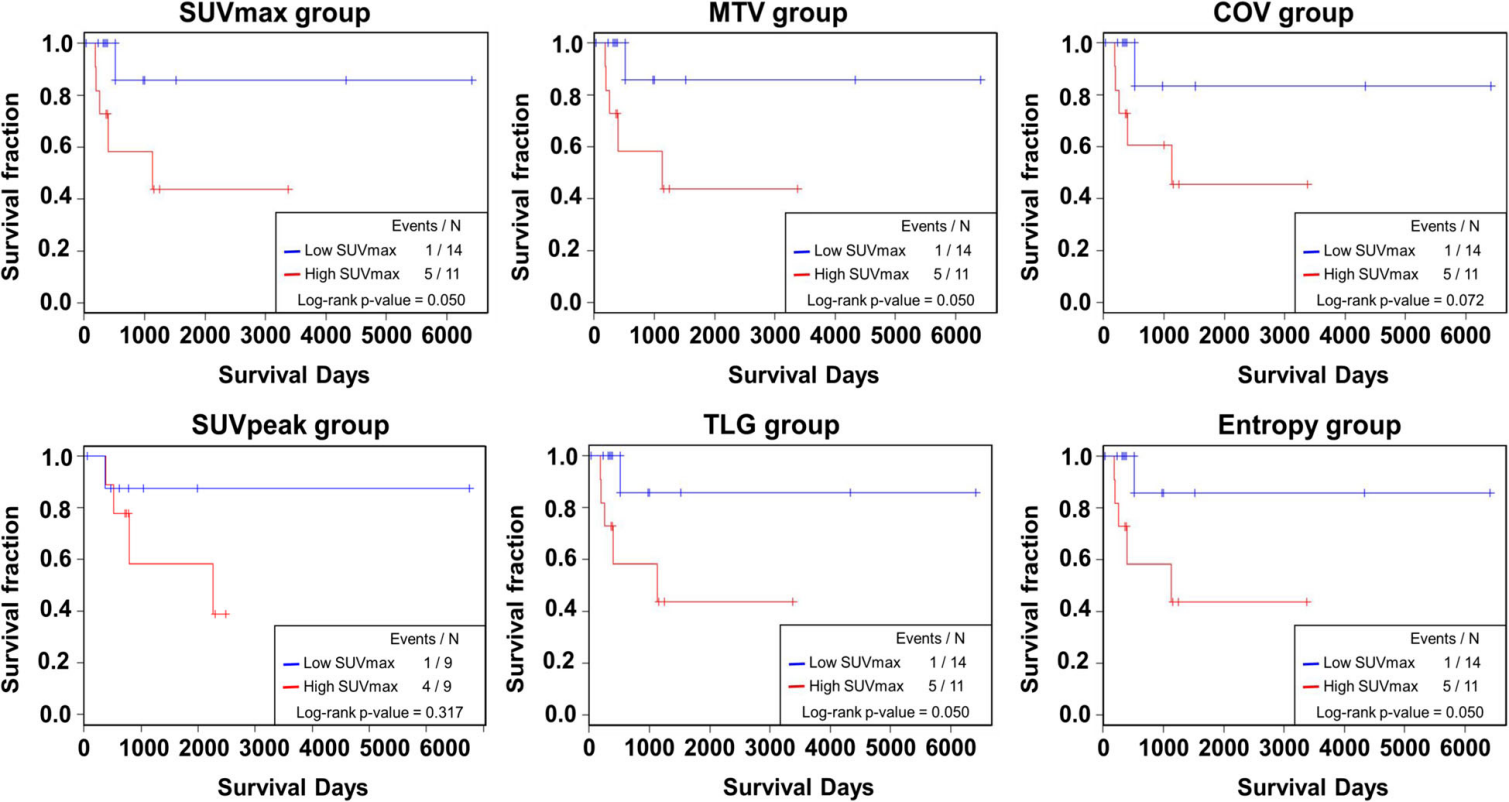
Prognostic value of FDG PET features. Kaplan-Meier curves of each group divided with adjusted cutoff value of FDG features. Survival analysis and log-rank test were performed to compare each group. Low and high FDG subsets for 25 patients. Red, high subset; blue, low subset. Of note, low and high MATH and GlycoS groups are divided according to the optimized cut-offs obtained by cut-off finder (http://molpath.charite.de/cutoff/index.jsp)

**Fig. 6:**
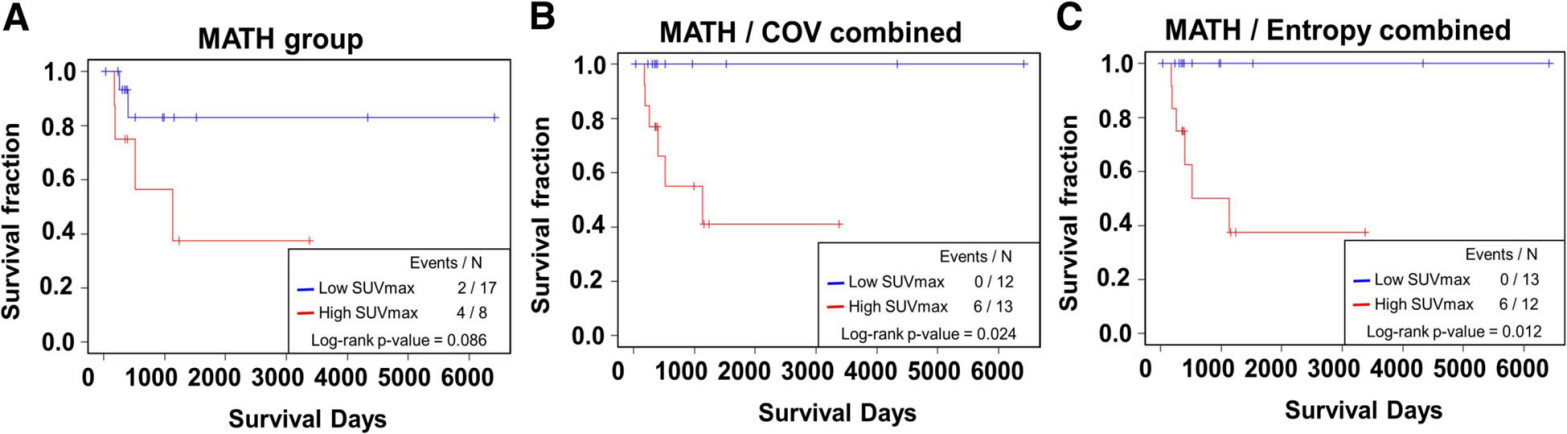
Predictive value of combined MATH and FDG PET features. Kaplan-Meier curves of each group divided with adjusted cutoff value of the features. **a** MATH showed a trend of prediction of OS (*P* = 0.086). **b, c** When MATH and FDG features were combined, the predictive value became more robust (B: MATH+COV, *P* = 0.024, C: MATH + Entropy, *P* = 0.012). Low group = patients in low group for both features; High group = patients in high group at least one feature. Of note, low and high groups are divided according to the optimized cut-offs obtained by cut-off finder (http://molpath.charite.de/cutoff/index.jsp)

We analyzed the prognostic value of genetic and FDG PET features. SUVmax, MTV, TLG, and entropy were predictive of OS (*P* < 0.05 for the features), while COV tended to predict the OS (*P* = 0.072) (Figs. 4 and 5). Also, MATH tended to predict the OS (*P* = 0.086) in the 25 patients. Thus, we tested the predictive value of using both heterogeneity features from genetic data and FDG PET. We divided the patients into two groups (low and high) based on both heterogeneity features. Low group consists of patients who are in low group for both MATH and FDG PET feature (COV or entropy) and high group consists of patients who are in high group for MATH and/or FDG PET feature (COV or entropy). We found that the combination of MATH and FDG heterogeneity features showed more robust predictive value of OS than using only MATH (MATH: *P* = 0.086, MATH + COV: *P* = 0.024, MATH + Entropy: *P* = 0.012, Fig. 6).

**Fig. 7:**
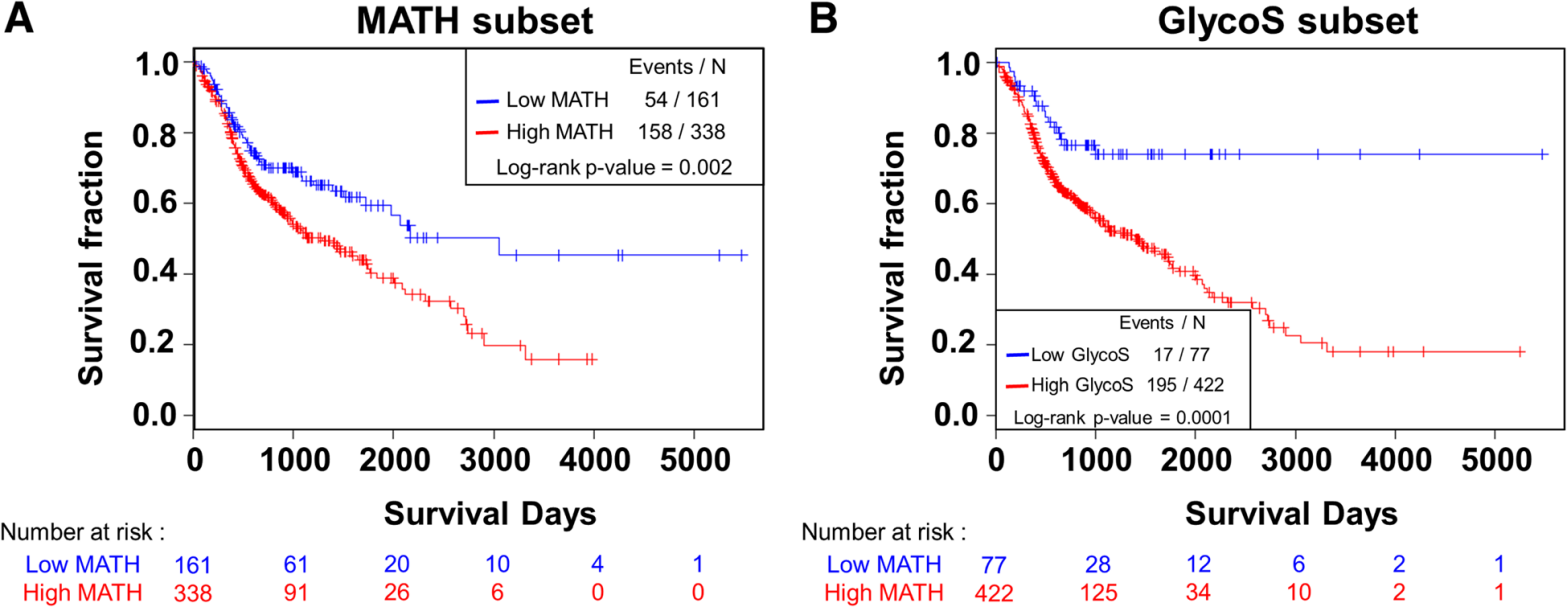
Prognostic value of MATH and GlycoS. Kaplan-Meier curves of each group divided with adjusted cutoff value of genetic signatures. Survival analysis and log-rank test were performed to compare each group. **a** Low MATH and high MATH subsets for 499 patients. Red, high MATH (MATH > 37.17); blue, low MATH (MATH < 37.17). **b** Same analysis as (**a**) comparing low GlycoS and high GlycoS subsets. Red, high GlycoS (GlycoS > 0.80); blue, low GlycoS (GlycoS < 0.80). Of note, low and high MATH and GlycoS groups are divided according to the optimized cut-offs obtained by cut-off finder (http://molpath.charite.de/cutoff/index.jsp)

Finally, we further analyzed the prognostic value of MATH and GlycoS in 499 patients. We found that MATH and GlycoS were highly predictive of OS in univariate analysis using log-rank test and Kaplan-Meyer analysis (*P* = 0.002 for MATH; *P* = 0.0001 for GlycoS) (Fig. 7a, b). MATH and GlycoS were predictive of OS even after adjustment using clinicopathologic features (age, sex, and tumor stage) in multivariate Cox regression analysis. Furthermore, both MATH and GlycoS were still significant prognostic factors even after including both features and the clinicopathologic features in the same model. This result indicates that both features have an additive role over each other to predict OS (*P* = 0.015 for MATH; *P* = 0.006 for GlycoS) (Table 2).

**Table 2:** Multivariate cox regression test for MATH and GlycoS

	Hazard ratio (95% CI)	*P* value
MATH (unadjusted)	1.013 (1.002–1.024)	0.016
MATH (adjusted for age, gender, and tumor stage)	1.016 (1.004–1.027)	0.007
MATH (adjusted for GlycoS, age, gender, and tumor stage)	1.014 (1.003–1.026)	0.015
GlycoS (unadjusted)	1.381 (1.129–1.689)	0.002
GlycoS (adjusted for age, gender, and tumor stage)	1.362 (1.112–1.668)	0.003
GlycoS (adjusted for MATH, age, gender, and tumor stage)	1.331 (1.085–1.633)	0.006

## Discussion

We found that the tumor metabolic features estimated by FDG PET showed a mild but statistically significant level of association with tumor genetic heterogeneity. Specifically, tumor metabolic-volumetric and metabolic heterogeneity features of FDG PET were associated with MATH in a mild degree. This finding supports the notion that quantifiable FDG uptake features reflect the tumor heterogeneity at the genomic level in HNSC [10]. Also, there was additive prognostic value when the FDG PET and genetic heterogeneity features were combined. Additionally, both genetic heterogeneity feature (MATH) and glycolysis feature (GlycoS) were independently predictive of OS even after adjusting for clinicopathologic features.

Recently, tumor imaging phenotypes were found to be related to gene expression profiles in HNSC [10, 17, 29–32]. Specifically, SUV and heterogeneity features estimated by FDG PET were related to 1177 differentially expressed genes in normal and tumor tissues [10]. Previous studies have shown a link between FDG uptake and several specific genes which modulate glucose metabolism [33–35]. Also, phenotypic whole tumor-level heterogeneity can be noninvasively recognized by various imaging techniques including computed tomography (CT), magnetic resonance imaging (MRI), and FDG PET [11]. However, it has been unclear whether the genetic heterogeneity assessed by a small sample of tumor tissue can reflect the whole tumor-level phenotypic heterogeneity or not [6]. Also, there has been no study to evaluate the association of tumor heterogeneity measured by FDG PET and genomic analysis in patients with HNSC. As cancer cells are evolved in a heterogeneous spatiotemporal environment based on genetic heterogeneity, we hypothesized that genetic heterogeneity might be associated with whole tumor level heterogeneity measured by FDG PET. In this study, by utilizing the database of TCGA and TCIA, we were able to find that there is an association between whole tumor level heterogeneity based on FDG PET and genetic heterogeneity in HNSC. Although the association was statistically significant, the level of association was weak with correlation coefficients of 0.4~0.5. This weak level of association was not a surprise because the methods to measure the tumor heterogeneity were totally different between FDG PET heterogeneity parameters and MATH. MATH was obtained from genetic sequencing data of a small portion of tumor, while FDG PET heterogeneity parameters were calculated from an imaging data reflecting metabolic status of a whole tumor area. It is noteworthy that there was a mild degree of association between MATH and FDG PET heterogeneity parameters, even with this striking difference of the methods to measure the tumor heterogeneity.

MATH is a genetic heterogeneity measure, which can be easily quantified as a percentage of mutant allele frequency among tumor-specific mutated loci. Also, the prognostic value of MATH has been validated in HNSC and colon cancer [9, 36, 37]. In the patients with HNSC, high MATH score was associated with increased mortality [36, 37]. Also, MATH was associated with the risk of metastases in patients with colon cancer [9]. However, the ability of MATH to represent tumor heterogeneity has not been tested by other modalities. We have demonstrated that MATH was highly associated with the representative heterogeneity features from FDG PET (entropy, COV). This result is in line with a recent study by Moon et al. They showed that Shannon’s heterogeneity index was associated with entropy in patients with small cell lung cancer [38]. Furthermore, we found that MATH was predictive of OS in patients with HNSC even after adjusting clinicopathologic features.

Recent meta-analyses showed that various FDG PET features including SUVmax, MTV, and TLG were prognostic factors in multiple types of malignancies [39–42]. Also, heterogeneity features of FDG PET have shown to be associated with treatment response and clinical outcome in multiple types of malignancies [13, 15, 43–45]. Among the heterogeneity features, entropy and COV have been widely accepted and proven to be useful for predicting treatment response and clinical outcomes [13, 15, 44]. For example, entropy was predictive of OS in pancreatic cancer [15], and the changes in entropy were independently associated with treatment response in erlotinib-treated non-small cell lung cancer [13]. Also, COV was superior to conventional parameters in predicting therapy response and disease progression in rectal cancer [44]. We found that entropy and COV were strongly associated with MATH, a genetic heterogeneity feature, which re-enforced the genetic background of the features and thus increased possibility of clinical utilization of the features.

MTV and TLG are radiomic features that represent metabolic-volumetric tumor burden. MTV is a measurement of tumor volume with increased glucose metabolism, while TLG is the product of MTV and the mean SUV of the volume. MTV and TLG are considered to be better prognostic factors than simple metabolic feature such as SUVmax [41, 46, 47]. In this study, we also found that MTV and TLG are significantly associated with genetic heterogeneity. As the tumor spatially grows, the larger volume of tumor likely to be more heterogenous reflecting genetic heterogeneity by cancer evolution. Multiple studies have shown that MTV and heterogeneity features of FDG PET such as COV and texture features are associated [48–50]. Therefore, tumor metabolic-volumetric features are likely to be an indicator of tumor genetic heterogeneity due to cancer evolution. Also, the association of MTV and TLG with genetic heterogeneity may further explain the robustness of the features in predicting the clinical outcomes.

Glycolysis is a crucial pathway regulating oncogenes, tumor suppressor genes, and glycolytic enzymes as well as accelerating cell proliferation in cellular metabolism [51]. Factors of metabolic glycolysis are associated with poor prognosis and tumor resistance to therapy in HNSC [52]. Also, glycolysis gene expression correlates FDG uptake features [30, 33–35]. However, the previous studies only explored the relationship of representative genes such as glucose transporter (GLUT) or hexokinase (HK). On the other hand, we utilized a novel glycolysis signature, GlycoS, which was derived from multiple glycolysis-associated genes defined by Reactome [19, 53]. We found that metabolic-volumetric features (MTV, TLG) were significantly associated with GlycoS. Unexpectedly, SUVmax and SUVpeak were not associated with GlycoS. One potential explanation is that many glycolysis-associated genes may not influence the intensity of FDG uptake, because the FDG uptake kinetics is primarily determined by glucose transportation by GLUT and phosphorylation by HK [54]. Even though, a higher number of patients may prove the associations between SUVmax and GlycoS, since there was a trend of positive correlation (*P* = 0.114). Also, we found that GlycoS is predictive of OS in patients with HNSC. Furthermore, GlycoS was predictive of OS even after adjusting MATH and clinicopathologic features. This implies that GlycoS has an additive prognostic value over MATH.

There are several limitations to this study. First, a limited number of samples were available in public archives. Also, we found only a mild degree of association between the genetic and FDG PET heterogeneity, and there were large number of scatters outside of the standard deviation (Fig. 2). Further studies will yield clearer results if analyzed using a larger number of expanded data. Second, FDG PET data from TCIA were applied to different technologies, reconstruction, and attenuation correction methods. So each image is difficult to compare to each other, and even SUVmax values vary, which may affect clinical decision [55]. To solve this problem, we did voxel interpolation to make all images have uniform voxel sizes. Also, we used entropy and COV as a tumor heterogeneity texture features because these are the most reliable upon reconstruction method. Third, although metabolic features and genomic signatures obtained in this study were candidates for future biomarkers, these are not validated precisely. Although we used representative genomic and metabolic features which have clinical implications with prognosis, there might be better features than these eight features. In a future study, more features could be considered for understanding cause and effect through systemic tumor biology. Nonetheless, our results show a correlation between genetic heterogeneity features and metabolic heterogeneity features and prognostic value about each feature.

## Conclusion

Tumor genetic heterogeneity was associated in a mild degree with metabolic heterogeneity measured by FDG PET in patients with HNSC. Genetic and metabolic heterogeneity features were predictive of OS, and there was additive prognostic value when the FDG PET and genetic heterogeneity features were combined. Moreover, genetic heterogeneity feature (MATH) and glycolysis feature (GlycoS) were independent predictors of OS.

## Data Availability

All data analyzed during this study are included in this manuscript.
